# USP1-Associated Factor 1 Modulates Japanese Encephalitis Virus Replication by Governing Autophagy and Interferon-Stimulated Genes

**DOI:** 10.1128/spectrum.03186-22

**Published:** 2023-03-29

**Authors:** Jinchao Xing, Chen Hu, Siqi Che, Yixin Lan, Lihong Huang, Lele Liu, Youqin Yin, Huanan Li, Ming Liao, Wenbao Qi

**Affiliations:** a College of Veterinary Medicine, South China Agricultural University, Guangzhou, China; b Key Laboratory of Zoonosis, Ministry of Agriculture and Rural Affairs, Guangzhou, China; c National and Regional Joint Engineering Laboratory for Medicament of Zoonosis Prevention and Control, Guangzhou, China; d Key Laboratory of Zoonoses Prevention and Control of Guangdong Province, Guangzhou, China; Regional Centre for Biotechnology

**Keywords:** interferon-stimulated gene, Japanese encephalitis virus, UAF1, autophagy

## Abstract

Japanese encephalitis virus (JEV) is a typical mosquito-borne flavivirus that can cause central nervous system diseases in humans and animals. Host factors attempt to limit virus replication when the viruses invade the host by using various strategies for replication. It is essential to clarify the host factors that affect the life cycle of JEV and explore its underlying mechanism. Here, we found that USP1-associated factor 1 (UAF1; also known as WD repeat-containing protein 48) modulated JEV replication. We found that JEV propagation significantly increased in UAF1-depleted Huh7 cells. Moreover, we found that knockdown of UAF1 activated cell autophagic flux in further functional analysis. Subsequently, we demonstrated that autophagy can be induced by JEV, which promotes viral replication by inhibiting interferon-stimulated gene (ISG) expression in Huh7 cells. The knockdown of UAF1 reduced ISG expression during JEV infection. To explore the possible roles of autophagy in UAF1-mediated inhibition of JEV propagation, we knocked out ATG7 to generate autophagy-deficient cells and found that depletion of UAF1 failed to promote JEV replication in ATG7 knockout cells. Moreover, in ATG7-deficient Huh7 cells, interference with UAF1 expression did not lead to the induction of autophagy. Taken together, these findings indicate that UAF1 is a critical regulator of autophagy and reveal a mechanism by which UAF1 knockdown activates autophagy to promote JEV replication.

**IMPORTANCE** Host factors play an essential role in virus replication and pathogenesis. Although UAF1 is well known to form complexes with ubiquitin-specific proteases, little is known about the function of the UAF1 protein itself. In this study, we confirmed that UAF1 is involved in JEV replication. Notably, we discovered a novel function for UAF1 in regulating autophagy. Furthermore, we demonstrated that UAF1 modulated JEV replication through its autophagy regulation. This study is the first description of the novel function of UAF1 in regulating autophagy, and it clarifies the underlying mechanism of the antiviral effect of UAF1 against JEV. These results provide a new mechanistic insight into the functional annotation of UAF1 and provide a potential target for increasing virus production during vaccine production.

## INTRODUCTION

Japanese encephalitis virus (JEV), which is in the genus *Flavivirus*, can cause acute encephalitis in humans and horses and reproductive failure in pigs (1, 2). Flaviviruses such as West Nile virus, Zika virus, dengue virus, yellow fever virus, and tick-borne encephalitis virus are important human pathogens. They have led to huge public health threats and economic losses around the world (3). Preventive vaccines against JEV are available, but there are no specific commercial anti-JEV drugs (4). Due to the shift in the JEV epidemic genotype, the current vaccine cannot fully protect recipients from the new genotype strain (5).

USP1-associated factor 1 (UAF1), which is also called WD repeat-containing protein 48, can bind USP1, USP12, and USP46 and stimulate the activity of these ubiquitin-specific proteases (USPs) (6). USP1, USP12, and USP46 are unified by their dependence on UAF1, which increases their activities by approximately 10- to 30-fold (7). UAF1 promotes USP46 protein abundance by binding to the deubiquitinating enzyme and inhibiting its ubiquitination (8). UAF1 deubiquitinase complexes enhance NLRP3 and pro-interleukin 1β (IL-1β) expression by targeting NLRP3 and p65 and licensing NLRP3 inflammasome activation (9). Moreover, the depletion of UAF1 and associated USPs reduces human papillomavirus (HPV) DNA replication (10–12). Herpesviruses can reportedly recruit UAF1 to downregulate T-cell receptor and CD4 surface expression, and herpesviruses may employ this mechanism to deregulate lymphocyte receptor expression to disarm host immune control (13). The USP1-UAF1 deubiquitinase complex facilitates TLR3/4-, RIG-I-, and cGAS-induced beta interferon (IFN-β) production to enhance antiviral responses during Sendai virus and vesicular stomatitis virus infection (14). However, the intrinsic functions and the antiviral effects of UAF1 are still unknown.

Autophagy is a highly conserved catabolic process that provides a self-digestion function (15). There is an extremely complex relationship between autophagy and viruses (16). An increasing number of studies show that viral infection activates autophagy. Autophagy functions as an intrinsic antiviral defense to restrict the replication of some viruses, including human immunodeficiency virus type 1 (17) and Rift Valley fever virus (18). However, some viruses, including influenza A virus (19) and enterovirus 71 (20), induce autophagy by manipulating the autophagic machinery to promote viral replication and pathogenesis. JEV infection also activates autophagy in infected cells (21). However, autophagy plays a dual role in regulating JEV replication (22–25). These studies imply that autophagy affects the JEV life cycle, but controversial observations about autophagy and JEV need to be further studied.

In this study, we investigated the role of UAF1 during JEV infection. We performed knockdown, knockout, and transcomplementation experiments to demonstrate the regulation of JEV replication by UAF1. Intriguingly, knocking down UAF1 activated autophagy and reduced interferon-stimulated-gene levels during JEV infection. Notably, this is the first report that UAF1 regulates JEV replication through autophagy. These findings warrant further studies to exploit novel functions of UAF1 and provide us with a better understanding of the JEV replication mechanism.

## RESULTS

### UAF1 inhibited the replication of JEV.

**FIG 1:**
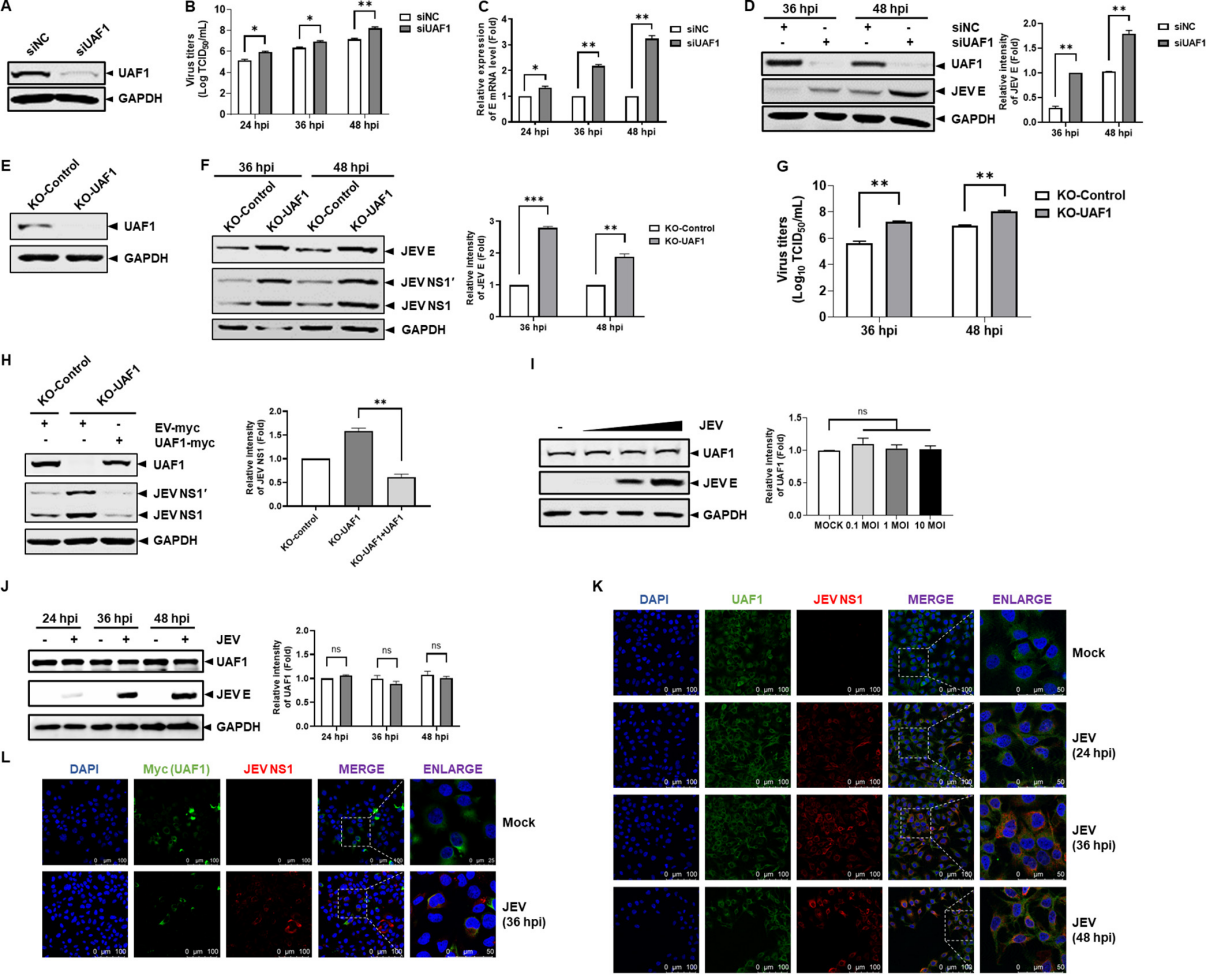
Knockdown of UAF1 promoted JEV replication. (A to D) Huh7 cells were transfected with NC siRNA (50 nM) or UAF1 siRNA (50 nM) for 36 h, followed by infection with JEV at an MOI of 0.1 for 24, 36, and 48 h. (A) The knockdown efficiency of UAF1 was examined by Western blotting. (B) Virus titers were subjected to TCID_50_ determination in BHK-21 cells. (C) JEV E mRNA levels were measured by real-time RT-PCR. (D) JEV E protein expression levels were detected by Western blotting. The relative intensity of JEV E protein expression levels was quantified with Image Studio V5.2. KO-UAF1 and KO-Control cells were infected with JEV at an MOI of 0.1 for 36 and 48 h. (E) The efficiency of the UAF1 knockout was examined by Western blotting. (F) JEV E and NS1 protein expression levels were detected by Western blotting. The relative intensity of JEV E protein expression levels was quantified with Image Studio V5.2. (G) Virus titers were subjected to TCID_50_ determination in BHK-21 cells. KO-UAF1 cells were transfected with pRE-UAF1 (1.5 μg) or pRE-empty (1.5 μg) plasmid for 36 h and then infected with JEV at an MOI of 0.1. Cells were collected at 36 hpi. (H) JEV NS1 protein expression levels were measured by Western blotting. The relative intensity of JEV NS1 protein expression levels was quantified by Image Studio V5.2. (I) Huh7 cells were infected with JEV at MOI of 0.01, 0.1, and 1. UAF1 protein expression levels were measured by Western blotting. The relative intensity of JEV UAF1 protein expression levels was quantified with Image Studio V5.2. (J) Huh7 cells were infected with JEV at an MOI of 0.1 and then were harvested at 24, 36, and 48 hpi. UAF1 protein expression levels were measured by Western blotting. The relative intensity of JEV UAF1 protein expression levels was quantified with Image Studio V5.2. (K) Confocal imaging of UAF1 distribution in JEV-infected cells immunostained with anti-UAF1 (green) and anti-NS1 (red) antibodies. Nuclei were labeled with DAPI (blue). Representative micrographic images are shown. Bars, 50 and 100 μm. (L) Huh7 cells were transfected with UAF1-myc plasmid and then infected with JEV at an MOI of 0.1. Confocal imaging of UAF1 distribution in JEV-infected cells immunostained with anti-Myc (green) and anti-NS1 (red) antibodies. Nuclei were labeled with DAPI (blue). Representative micrographic images are shown. Bars, 25 and 100 μm. Data from one experiment of three are shown. The data were analyzed with Prism 7 software (GraphPad Software) using a two-tailed Student's *t* test (ns, not significant; *, *P* < 0.05; **, *P* < 0.01; ***, *P* < 0.001).

To explore the involvement of endogenous UAF1 in regulating JEV replication, UAF1 knockdown with small interfering RNA (siRNA) was performed in Huh7 cells. Huh7 cells were transfected with UAF1 or nontargeting control (NC) siRNA. As shown in Fig. 1A, UAF1 protein expression in the UAF1 siRNA-transfected cells was markedly decreased at 36 h posttransfection (hpt). At 36 hpt, the knockdown cells were infected with JEV. Supernatants and cells were harvested at 24, 36, and 48 h postinfection (hpi). Viral titers and levels of RNA and E protein expression were determined by measurement of the 50% tissue culture infection dose (TCID_50_), real-time reverse transcription-PCR (RT-PCR), and Western blotting, respectively. The viral titer was approximately 10-fold higher in UAF1 knockdown cells than in control knockdown cells (Fig. 1B). UAF1 knockdown also significantly increased viral mRNA and protein levels (Fig. 1C and D). The results showed that knocking down UAF1 significantly promoted the replication of JEV.

To confirm our finding that UAF1 knockdown can modulate the replication of JEV, we established UAF1 knockout (KO-UAF1) and control knockout (KO-Control) Huh7 cell lines by using the CRISPR/Cas9 system. The effect of UAF1 knockout was assessed by Western blotting. As shown in Fig. 1E, endogenous UAF1 protein expression was abrogated in the KO-UAF1 cell line. Subsequently, KO-UAF1 and KO-Control cells were infected with equal amounts of JEV for 36 and 48 h. The viral proteins and virus titers were measured by Western blotting. As expected, the depletion of UAF1 extensively promoted viral protein expression and infectious virion production (Fig. 1F and G).

We further evaluated the antiviral effect of UAF1 transcomplementation on JEV replication in KO-UAF1 cells. KO-UAF1 cells were transfected with pRE-UAF1 or pRE-empty vector, and then cells were infected with equal amounts of JEV. The rescue of UAF1 significantly decreased viral NS1 protein expression (approximately 3-fold lower than in the empty plasmid transfection group) in KO-UAF1 cells (Fig. 1H). Collectively, these results indicated that UAF1 inhibited JEV replication.

Subsequently, changes in UAF1 protein expression were explored during JEV infection in Huh7 cells. Huh7 cells were infected with JEV at different multiplicities of infection (MOI) or infected with JEV at different times. The results showed that JEV infection did not affect UAF1 protein expression (Fig. 1I and J). Simultaneously, we observed intracellular localization of UAF1 during JEV infection. The results showed that endogenous UAF1 protein was present in the cytoplasm, and JEV infection did not alter its localization (Fig. 1K). We overexpressed UAF1 plasmids in Huh7 cells, and the results were the same as those of endogenous UAF1 localization (Fig. 1L). These data demonstrated that JEV did not affect UAF1 localization.

### Knockdown of UAF1 induced cell autophagic flux.

**FIG 2:**
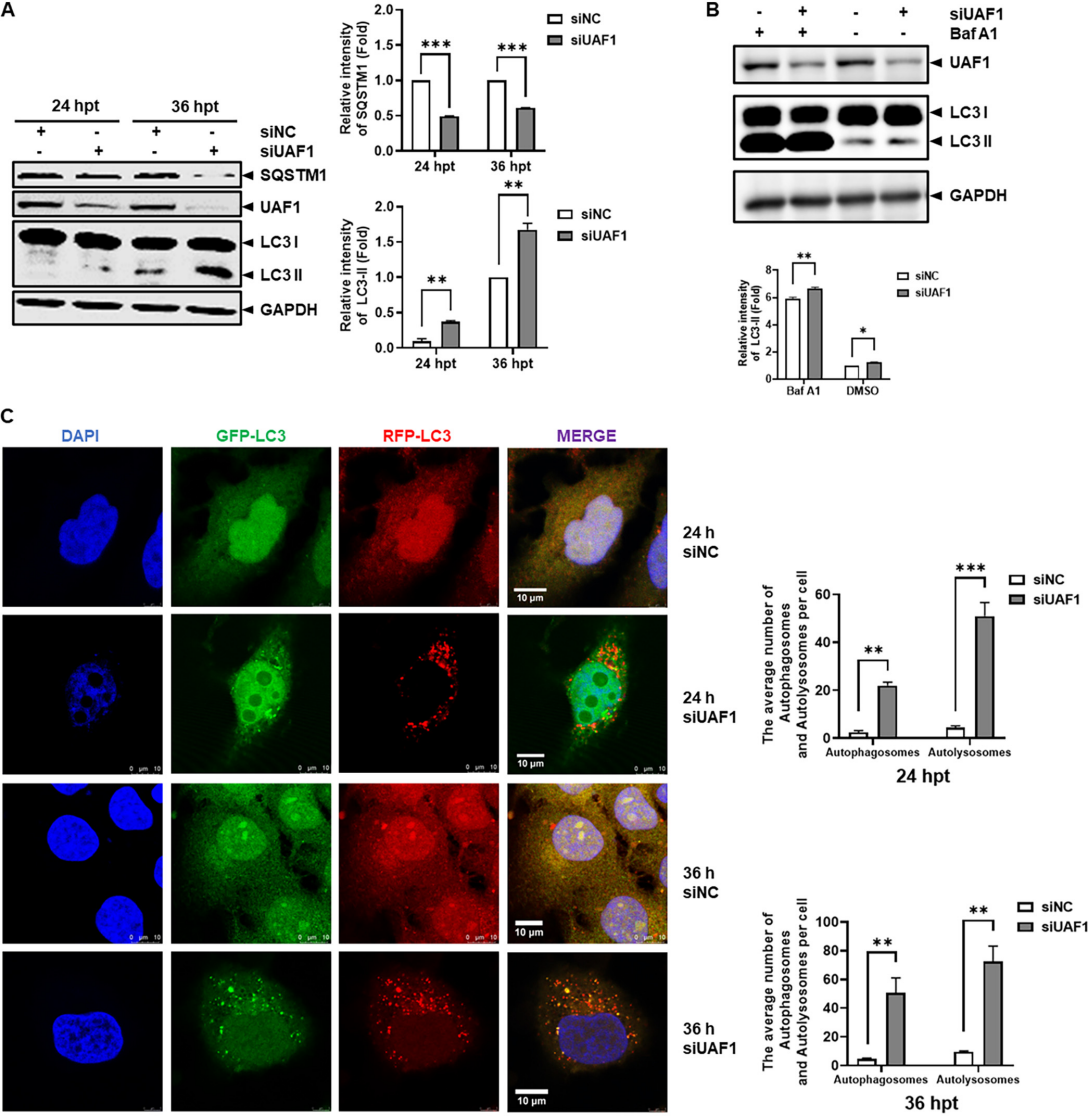
Knockdown of UAF1 activated autophagy. (A) Huh7 cells were transfected with NC siRNA or UAF1 siRNA for 24 and 36 h. The UAF1, SQSTM1, and LC3 protein expression levels were detected by Western blotting. The relative intensities of SQSTM1 and LC3-II protein expression levels were quantified by Image Studio V5.2. (B) Huh7 cells were transfected with NC siRNA or UAF1 siRNA for 24 h and then treated with bafilomycin A1 (BafA1; 50 nM) for 12 h. LC3 protein expression levels were detected by Western blotting. The relative intensities of LC3-II protein expression levels were quantified by Image Studio V5.2. (C) GFP-RFP-LC3 Huh7 cells were seeded on coverslips in 24-well plates and transfected with UAF1 siRNA or NC siRNA for 24 h and 36 h. The cells were observed by confocal microscopy. Bar, 10 μm. The graph shows the quantification of autophagosomes and autolysosomes by taking the average number of dots in 50 cells. The data were analyzed with Prism 7 software (GraphPad Software) using a two-tailed Student's *t* test (*, *P* < 0.05; **, *P* < 0.01; ***, *P* < 0.001).

Previous studies have indicated that the UAF1 protein primarily regulates DNA damage repair and interacts with USP1, USP12, and USP46 to catalyze the deubiquitinating activity of these proteins (26). Our study found that the UAF1 protein can modulate the cell autophagy process. Huh7 cells were transfected with UAF1 siRNA or NC siRNA, and cells were harvested at 24 and 36 hpt. As shown in Fig. 2A, UAF1 knockdown significantly reduced SQSTM1 protein expression and increased LC3-II protein expression. Furthermore, we used bafilomycin A1 (a standard method to monitor functional autophagy flux) to monitor the impact of UAF1 deficiency on autophagy. Using bafilomycin A1, we found that UAF1 knockdown increased the accumulation of LC3-II (Fig. 2B).

To study the autophagy mechanism regulated by UAF1, we established Huh7 cells that stably expressed GFP-RFP-LC3 to investigate their autophagic flux. When autophagy is activated, scattered LC3 forms autophagosomes. At the late stage of autophagy, autophagosomes and lysosomes fuse to form autolysosomes. Autolysosomes exhibit only red fluorescence, because green fluorescence is sensitive to the acidic conditions in the lysosomal lumen and can be quenched. Huh7 cells expressing green fluorescent protein, red fluorescent protein, and LC3 (GFP-RFP-LC3 Huh7 cells) seeded on coverslips in 24-well plate were transfected with UAF1 siRNA or NC siRNA. The cells were fixed and stained with 4,6-diamidino-2-phenylindole (DAPI) at 24 and 36 hpt. The results of laser scanning confocal microscopy showed that the number of autophagosomes (green particles) in the UAF1-silenced cells significantly increased, and the same tendency occurred for autolysosomes (red particles) (Fig. 2C). Taken together, these data demonstrated that UAF1 played an important role in autophagy regulation.

### JEV infection activated autophagy to promote virus replication.

**FIG 3:**
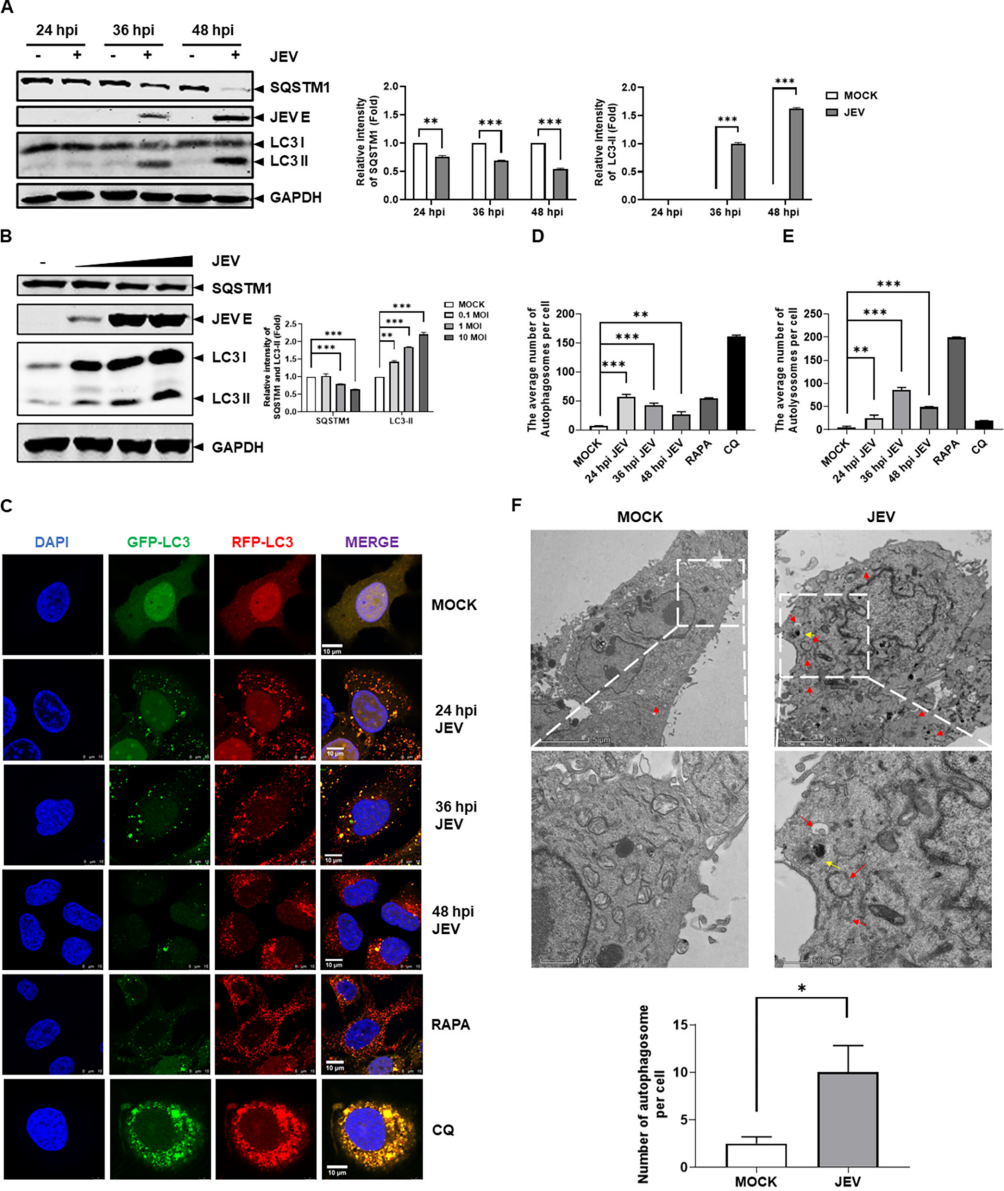
JEV infection enhanced autophagic flux. (A) Huh7 cells were infected with JEV at an MOI of 0.1 for 24, 36, and 48 h. JEV E, SQSTM1, and LC3 protein expression levels were measured by Western blotting. The relative intensities of SQSTM1 and LC3-II protein expression levels were quantified by Image Studio V5.2. (B) Huh7 cells were mock infected or infected with JEV at MOI of 0.1, 1, and 10 for 36 h. The JEV E, SQSTM1, and LC3 protein expression levels were detected by Western blotting. The relative intensities of SQSTM1 and LC3-II protein expression levels were quantified by Image Studio V5.2. (C) GFP-RFP-LC3 Huh7 cells seeded on coverslips in a 24-well plate were infected with JEV at an MOI of 0.1 for 24, 36, and 48 h. GFP-RFP-LC3 Huh7 cells as well as the RAPA (500 nM) and CQ (10 μM) groups were used as controls. The cells were observed by confocal microscopy. Bar, 10 μm. (D and E) Quantification of autophagosomes (D) and autolysosomes (E) by taking the average number of dots in 50 cells. (F) Huh7 cells were infected with JEV at an MOI of 1 for 36 h and observed by electron microscopy. Red arrows indicate the structures with autophagosomes. Yellow arrows indicated the structures with the autolysosomes. Bar, 2 μm. The data were analyzed with Prism 7 software (GraphPad Software) using a two-tailed Student's *t* test (*, *P* < 0.05; **, *P* < 0.01; ***, *P* < 0.001).

As shown above, a novel function of UAF1 has been demonstrated to regulate autophagy. The mechanism by which UAF1 regulates JEV replication is still unknown. These results prompted us to consider whether UAF1 modulates JEV replication through autophagy. We first explored changes in autophagy during JEV infection. Huh7 cells were infected with JEV and were then collected at 24, 36, and 48 hpi, and the cell lysates were measured by Western blotting. The basal protein expression levels of SQSTM1 and LC3-II in the mock-infected cells were assessed. JEV infection significantly changed SQSTM1 and LC3-II protein expression in Huh7 cells at 36 and 48 hpi (Fig. 3A). Subsequently, Huh7 cells were mock infected or infected with JEV at different MOI and collected at 36 hpi. The cell lysates were measured by Western blotting. The expression levels of SQSTM1 and LC3-II proteins were significantly decreased and upregulated during JEV infection (Fig. 3B). Simultaneously, a confocal experiment was used to observe changes in autophagic flux in GFP-RFP-LC3 Huh7 cells during JEV infection. GFP-RFP-LC3 Huh7 cells were infected with JEV and then fixed and stained with DAPI. Rapamycin (RAPA) is a well-known autophagy agonist that can activate autophagy (27, 28). Chloroquine (CQ), originally identified as an inhibitor of endocytosis (29), is an autophagy inhibitor that works by regulating the acidic cavity conditions in the autolysosome lumen (30). RAPA and CQ were used as positive controls. Confocal observation showed that the number of autophagosomes and autolysosomes induced by JEV increased significantly (Fig. 3C to E). Furthermore, we explored the formation of autophagosome vesicles during JEV infection by transmission electron microscopy (TEM). The results showed that double- or single-membrane vesicles were induced by JEV (Fig. 3F). These findings showed that JEV could activate autophagy.

**FIG 4:**
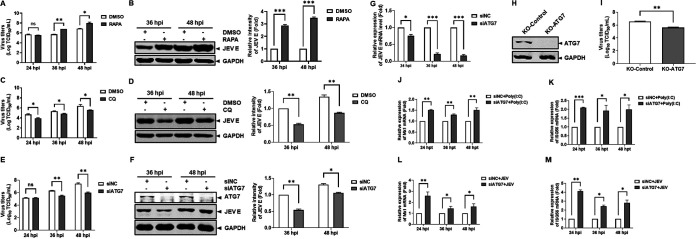
Autophagy positively modulated JEV replication. Huh7 cells were pretreated with RAPA (500 nM) for 2 h and then infected with JEV at an MOI of 0.1 for 24, 36, and 48 h. (A) Virus titers were subjected to TCID_50_ determination in BHK-21 cells. (B) JEV E protein expression levels were detected by Western blotting. The relative intensity of JEV E protein expression levels was quantified by Image Studio V5.2. Huh7 cells were pretreated with CQ (10 μM) for 2 h and then infected with JEV at an MOI of 0.1 for 24, 36, and 48 h. The samples were subjected to virus titer determination (C) and Western blotting (D). The relative intensity of JEV E protein expression levels was quantified by Image Studio V5.2. Huh7 cells were transfected with NC siRNA (50 nm) or ATG7 siRNA (50 nm) for 36 h, followed by infection with JEV at an MOI of 0.1 for 24, 36, and 48 h. (E) Virus titers were subjected to TCID_50_ determination in BHK-21 cells. (F) JEV E protein expression levels were detected by Western blotting. The relative intensity of JEV E protein expression levels was quantified by Image Studio V5.2. (G) JEV E mRNA levels were measured by real-time RT-PCR. KO-ATG7 and KO-Control cells were infected with JEV at an MOI of 0.1 for 36 h. (H) The knockout efficiency of ATG7 was examined by Western blotting. (I) Virus titers were subjected to TCID_50_ on BHK-21 cells. Huh7 cells were transfected with NC siRNA or ATG7 siRNA for 36 h, followed by transfection with 3 μg/mL poly(I·C) for 24, 36, and 48 h. Mx1 (J) and ISG56 (K) mRNA levels were measured by real-time RT-PCR. Huh7 cells were transfected with NC siRNA or ATG7 siRNA for 36 h, followed by infection with JEV at an MOI of 0.1 for 24, 36, and 48 h. Mx1 (L) and ISG56 (M) mRNA levels were measured by real-time RT-PCR during JEV infection. Data from one experiment of three are shown. The data were analyzed with Prism 7 software (GraphPad Software) using a two-tailed Student's *t* test (ns, not significant; *, *P* < 0.05; **, *P* < 0.01; ***, *P* < 0.001).

Subsequently, we investigated the role of autophagy in JEV replication. We chose to use the autophagy activator RAPA. Huh7 cells were pretreated with RAPA or dimethyl sulfoxide (DMSO) for 2 h before JEV infection. The viral titer and viral protein expression were tracked by TCID_50_ measurement and Western blotting. We found that RAPA significantly increased the viral titers at 36 and 48 hpi (Fig. 4A). Western blotting showed that RAPA significantly increased viral E protein expression, approximately 3-fold (Fig. 4B). Correspondingly, Huh7 cells were pretreated with CQ for 2 h before JEV infection. The results showed that CQ significantly restricted JEV replication in Huh7 cells (Fig. 4C and D).

CQ is an endocytosis inhibitor and autophagy inhibitor. To exclude the possibility that CQ restricted JEV replication through endocytosis, we silenced autophagy-related genes. ATG7 plays an essential role in the formation of autophagosomes (31). Huh7 cells transfected with ATG7 siRNA or NC siRNA were infected with JEV at 36 hpt. Virus yields, viral E protein levels, and viral RNA levels were measured. As shown in Fig. 4E to G, ATG7 knockdown caused remarkable decreases in the viral titer (approximately 25-fold lower at 48 hpi), viral E protein expression, and viral RNA (approximately 80% lower at 36 and 48 hpi). We established ATG7 knockout (KO-ATG7) Huh7 cell lines by using the CRISPR/Cas9 system. As expected, the viral titer decreased in KO-ATG7 cells compared to KO-Control cells (Fig. 4H and I). In short, these data proved that JEV activated autophagy to promote its replication in Huh7 cells.

The innate immune response is the first line of host defense against virus invasion. However, some viruses weaken immune defenses by cooperating with autophagy (32). To investigate the mechanisms behind the modulation of autophagy, the interferon-stimulated-gene (ISG) levels were monitored by real-time RT-PCR analysis. After ATG7 silencing, we examined the ISG mRNA levels. The Mx1 and ISG56 mRNA levels were increased in ATG7-silenced cells compared to the control cells (Fig. 4J and K). Strikingly, the Mx1 and ISG56 mRNA levels were further increased in ATG7-silenced cells during JEV infection (Fig. 4L and M). These data suggested that autophagy negatively modulated ISG activation.

### Depletion of UAF1 promoted autophagy and decreased ISG levels during JEV infection.

**FIG 5:**
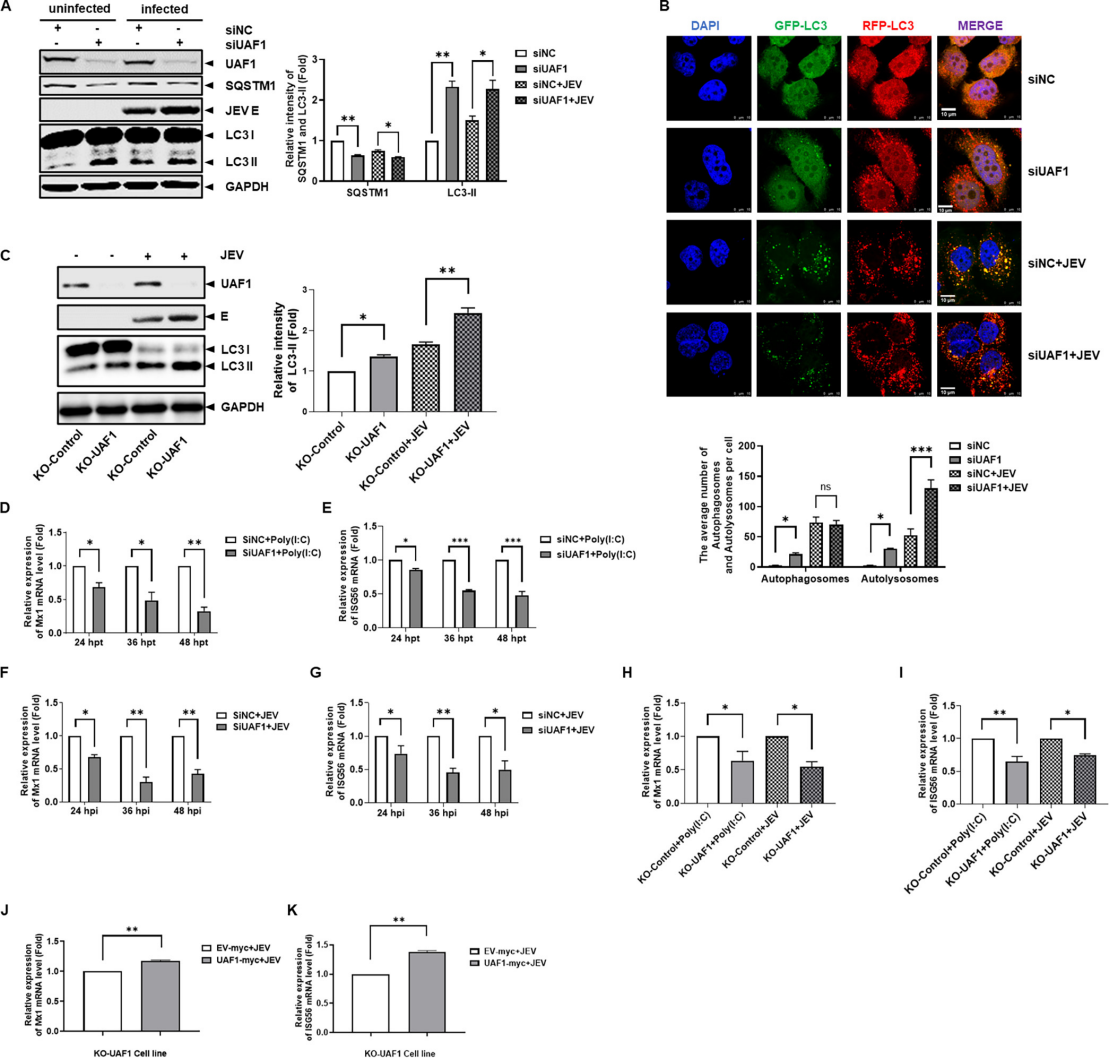
Knockdown of UAF1 enhanced autophagy and reduced ISG levels during JEV infection. (A) Huh7 cells were transfected with NC siRNA or UAF1 siRNA for 36 h, followed by mock infection or infection with JEV at an MOI of 0.1 for 36 h. UAF1, SQSTM1, and LC3 protein expression levels were detected by Western blotting. The relative intensities of SQSTM1 and LC3-II protein expression levels were quantified by Image Studio V5.2. (B) GFP-RFP-LC3 Huh7 cells were seeded on coverslips in 24-well plates and transfected with UAF1 siRNA or NC siRNA for 36 h, followed by mock infection or infection with JEV at an MOI of 0.1 for 36 h. The cells were observed by confocal microscopy. Bar, 10 μm. The graph shows the quantification of autophagosomes and autolysosomes by taking the average number of dots in 50 cells. (C) KO-Control cells and KO-UAF1 cells were mock infected or infected with JEV at an MOI of 0.1 for 36 h. LC3 protein expression levels were detected by Western blotting. The relative intensities of LC3-II protein expression levels were quantified by Image Studio V5.2. Huh7 cells were transfected with NC siRNA or UAF1 siRNA for 36 h, followed by transfection with 3 μg/mL poly(I·C) for 24, 36, and 48 h. Mx1 (D) and ISG56 (E) mRNA levels were measured by real-time RT-PCR. Huh7 cells were transfected with NC siRNA or UAF1 siRNA for 36 h, followed by infection with JEV at an MOI of 0.1 for 24, 36, and 48 h. Mx1 (F) and ISG56 (G) mRNA levels were measured by real-time RT-PCR during JEV infection. KO-Control cells and KO-UAF1 cells were transfected with poly(I·C) or infected with JEV at an MOI of 0.1 for 36 h. Mx1 (H) and ISG56 (I) mRNA levels were tested by real-time RT-PCR. KO-UAF1 cells were transfected with EV-myc or UAF1-myc plasmids and then infected with JEV at an MOI of 0.1 for 36 h. Mx1 (J) and ISG56 (K) mRNA levels were tested by real-time RT-PCR. Data from one experiment of three are shown. The data were analyzed with Prism 7 software (GraphPad Software) using a two-tailed Student's *t* test (ns, not significant; *, *P* < 0.05; **, *P* < 0.01; ***, *P* < 0.001).

We have demonstrated that UAF1 knockdown can activate autophagy. However, the association of UAF1 with autophagy remained unclear in virus-infected cells. Therefore, further studies focused on the effects of UAF1 on autophagy during JEV infection. Huh7 cells transfected with UAF1 siRNA or NC siRNA were mock infected or infected with JEV. Western blotting showed that UAF1 knockdown significantly reduced SQSTM1 protein expression and increased LC3-II protein expression during JEV infection (Fig. 5A). GFP-RFP-LC3 Huh7 cells were transfected with UAF1 siRNA or NC siRNA and were mock infected or infected with JEV. Then, the cells were fixed and stained with DAPI at 36 hpi. Consistently, the confocal experiment showed that UAF1 knockdown enhanced autolysosome accumulation during JEV infection (Fig. 5B). Subsequently, we explored the level of basal/induced autophagy in the normal and infected state in the UAF1 knockout condition. KO-UAF1 cells and KO-Control cells were mock infected or infected with JEV. LC3-II protein levels were increased in KO-UAF1 cells (Fig. 5C). Taken together, these data showed that the depletion of UAF1 enhanced JEV-induced autophagy.

Considering the relationship between UAF1 and autophagy, we examined the changes in ISG mRNA levels in UAF1-silenced cells. The Mx1 and ISG56 mRNA levels were significantly reduced in UAF1-silenced cells during mock or JEV infection (Fig. 5D to G). Subsequently, we observed that the ISGs levels were also decreased in KO-UAF1 cells (Fig. 5H and I). Transcomplementation expression of UAF1 significantly increased ISG levels in KO-UAF1 cells (Fig. 5J and K). These results indicated that UAF1 depletion inhibited the ISG levels.

### UAF1 depletion promoted JEV replication through autophagy.

**FIG 6:**
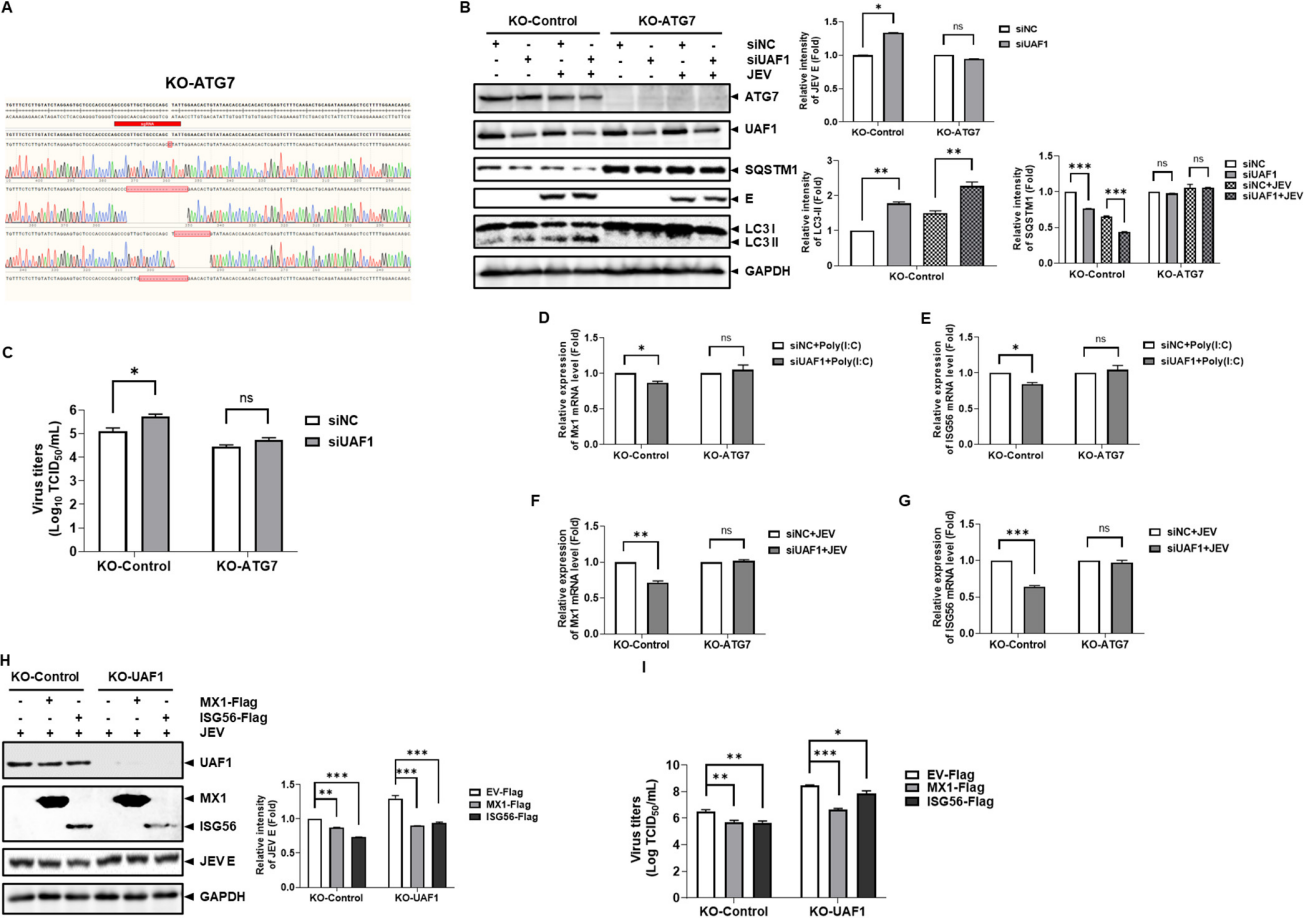
The changes in JEV replication and ISG levels were blocked after UAF1 silencing in KO-ATG7 cells during JEV infection. (A) Sequencing confirmed the genomic ablation of the ATG7 genes. Cells genomic DNA were harvested and the sgRNA region was amplified by PCR. (B and C) KO-ATG7 and KO-Control cells were transfected with NC siRNA or UAF1 siRNA for 36 h, followed by infection with JEV at an MOI of 0.1 for 36 h. (B) JEV E, ATG7, UAF1, SQSTM1, and LC3 protein expression levels were detected by Western blotting. The relative intensity of protein expression levels was quantified with Image Studio V5.2. (C) Virus titers were subjected to TCID_50_ determination in BHK-21 cells. KO-ATG7 and KO-Control cells were transfected with NC siRNA or UAF1 siRNA for 36 h, followed by transfection with 3 μg/mL poly(I·C) for 36 h. Mx1 (D) and ISG56 (E) mRNA levels were measured by real-time RT-PCR. KO-ATG7 and KO-Control cells were transfected with NC siRNA or UAF1 siRNA for 36 h, followed by infection with JEV at an MOI of 0.1 for 36 h. Mx1 (F) and ISG56 (G) mRNA levels were measured by real-time RT-PCR during JEV infection. KO-Control cells and KO-UAF1 cells were transfected with EV-Flag, Mx1-Flag, or ISG56-Flag plasmids and then infected with JEV at an MOI of 0.1 for 48 h. (H) JEV E protein expression levels were detected by Western blotting. (I) Virus titers were subjected to TCID_50_ in BHK-21 cells. Data from one experiment of three are shown. The data were analyzed with Prism 7 software (GraphPad Software) using a two-tailed Student's *t* test (ns, not significant; *, *P* < 0.05; **, *P* < 0.01; ***, *P* < 0.001).

The knockdown of UAF1 led to stronger autophagy and viral replication in JEV-infected cells. Subsequently, we explored whether the enhanced effect of UAF1 silencing on viral replication could be blocked by autophagy. We used an independent set of KO-Control and KO-ATG7 cells. Our sequencing results confirmed the genomic ablation of the targeted ATG7 genes (Fig. 6A). KO-ATG7 cells and KO-Control cells were transfected with UAF1 siRNA or NC siRNA. At 36 hpt, the knockdown cells were infected with JEV. The supernatants and cells were harvested at 36 hpi. The viral titers and the intracellular viral protein expression were determined by TCID_50_ measurement and Western blotting, respectively. We found that UAF1 knockdown increased viral replication in KO-Control cells (Fig. 6B and C). Interestingly, UAF1 knockdown did not significantly change viral replication in KO-ATG7 cells (Fig. 6B and C). It did enhance autophagy and JEV replication, and this enhancement of JEV replication was blocked by autophagy. These observations illustrated that UAF1 modulated JEV replication through autophagy.

Autophagy negatively modulated ISG activation. Furthermore, UAF1 knockdown inhibited ISG levels. These results prompted us to speculate that the regulation of ISG levels by UAF1 is due to autophagy. To confirm this hypothesis, KO-ATG7 cells and KO-Control cells were transfected with UAF1 siRNA or NC siRNA. No obvious changes in Mx1 and ISG56 levels were observed after UAF1 silencing in KO-ATG7 cells (Fig. 6D and E). Moreover, the changes in Mx1 and ISG56 levels were also blocked after UAF1 silencing in the KO-ATG7 cells during JEV infection (Fig. 6F and G).

To confirm that ISGs participated in virus replication in a UAF1-driven manner, we overexpressed ISGs in UAF1 knockout cells. KO-Control cells and KO-UAF1 cells were transfected with Mx1-Flag and ISG56-Flag plasmids and then were infected with JEV. The results showed that ISGs suppressed JEV replication (Fig. 6H and I).

Overall, these results showed that UAF1 modulated JEV replication through the ISG levels induced by autophagy. All these data strongly underscored the idea that UAF1 modulates JEV replication through its newly identified function of autophagy regulation.

## DISCUSSION

Cellular factors and pathways are often hijacked and exploited by viruses for their replication (33). In this study, we verified that UAF1 modulated JEV infection and uncovered a novel function of UAF1 in autophagy regulation.

There are few studies on UAF1 and viruses, and they are mostly focused on DNA viruses, such as HPV and herpesviruses, which play their key roles through the UAF1-USP deubiquitinase complex (34–37). In RNA viruses, the UAF1/USP1 deubiquitinase complex stabilizes TBK1 and enhances antiviral responses during Sendai virus and vesicular stomatitis virus infection (14). However, little is known about the regulatory role of UAF1 during flavivirus infection. In this research, we demonstrated that UAF1 knockdown and knockout promoted JEV replication. The rescue of UAF1 significantly suppressed JEV replication in KO-UAF1 cells. However, JEV infection did not alter UAF1 protein expression and localization. These results indicated that UAF1 suppressed JEV replication.

As the cofactor of USP1, USP12, and USP46, UAF1 could enhance their deubiquitinase activity by forming stable UAF1/USP protein complexes (6, 38). USP1 can regulate the Fanconi anemia pathway, but this regulation requires the formation of a stable UAF1/USP1 protein complex (39, 40). The UAF1/USP1 deubiquitinase complex selectively removes K48-linked polyubiquitination of NLRP3 to enhance cellular NLRP3 levels (9). The UAF1/USP12 complex deubiquitinates PHLPP1 and suppresses the proliferation of tumor cells (41). USP46 has almost no deubiquitination activity and needs to interact with UAF1 to achieve high activity (6, 38). UAF1/USP12 and UAF1/USP46 complexes promote NF-κB activation and enhance the transcription of NLRP3 and proinflammatory cytokines (9). Yu et al. (14) found that USP1-UAF1 deubiquitinase complex stabilizes TBK1 and enhances antiviral responses. ML323 (a specific USP1-UAF1 inhibitor) and USP1/UAF1 knockdown attenuated IFN-β expression and enhanced viral replication both *in vitro* and *in vivo* (14). However, we found that ML323 treatment and USP1 knockdown could restrict JEV replication (data not shown). These results differ from those of Yu et al. Simultaneously, our results showed that ML323 with USP1 and UAF1 play different roles in the JEV life cycle. The specific mechanism of ML323 and USP1 in the JEV life cycle still needs further exploration. In this study, we clarify an utterly novel role for UAF1. UAF1 knockdown increased LC3-II protein expression and reduced SQSTM1 protein expression. In GFP-RFP-LC3 Huh7 cells, confocal experiments showed that UAF1 knockdown induces cell autophagic flux. Together, these data demonstrated that UAF1 played an important role in autophagy regulation.

Autophagy is a cellular recycling mechanism for recovering nutrients to regenerate organelles and energy from cellular waste components (42, 43). Autophagy consists of a series of sequential processing events, such as initiation, isolation membrane elongation and nucleation, autophagosome cargo recruitment and maturation, autophagosome transportation, and the docking and fusion of the autophagosome with late endosomes or lysosomes (44). These events are critically coordinated by the actions of a range of key components, including autophagy-associated proteins (ATGs), and mediated by complex networks, such as the mechanistic target of rapamycin (mTOR), a major regulator of autophagy, and the mTOR-independent signaling pathway (45). Among mTOR-independent pathways, calcium ion signaling and endoplasmic reticulum (ER) stress are important signaling pathways regulating autophagy (45). Moreover, JEV-induced autophagy activation has been proved to be dependent on XBP1 and ATF6 ER stress sensors in neuronal cells (25). Further research is needed to explore whether UAF1 depletion regulates the occurrence of autophagy through XBP1 and ATF6 ER stress sensors.

Autophagy is an evolutionarily conserved catabolic cellular process that performs antiviral functions during viral invasion. However, coevolution between viruses and autophagy has allowed viruses to hijack autophagosomes as replication sites, or to hijack secreted autophagy pathways to promote maturation of viral particles and thus increase virus amplification (46). Li et al. reported that the autophagy inhibitor 3-MA suppresses the growth of JEV (47). Then, another research group found that knocking down ATG5 and Beclin 1 inhibited JEV replication by enhancing cell apoptosis and antiviral immunity (48). The loss of SQSTM1 from mouse embryonic fibroblasts restricts JEV replication (49). Previously, we reported that JEV utilizes the lysosomes membrane for RNA replication and hijacks the autophagic machinery to benefit virus replication (50). It has been reported that JEV NS4A induces mitophagy to promote viral infection, and inhibiting either mitochondrial fragmentation or mitophagy impairs virus propagation (51). The autophagy inhibitors reduce JEV infection and weaken the inflammatory response in mice (52). In contrast, the E3 ubiquitin ligase Nedd4 promotes JEV replication by suppressing autophagy in human neuroblastoma cells (24). Sharma and colleagues demonstrated that JEV replication is significantly enhanced in neuronal cells and ATG5-deficient mouse embryonic fibroblasts (53). Pharmacological induction of ER stress further activates autophagy and reduced JEV replication (25). However, the exact role of autophagy in JEV infection remains unclear (54). Analogous to JEV, autophagy differentially regulates the replication of Dengue virus and Zika virus in different infectious cell models (55, 56). The controversial results may be caused by different virus strains in different cells with different effects on virus replication (22–25). Whether autophagy promotes or inhibits viral replication is uncertain and depends on several factors, including the type of cell infected, the virus strain, and the infection conditions (57). In the present study, we demonstrated that JEV could induce autophagy. We used RAPA to demonstrate that autophagy can positively regulate viral replication in Huh7 cells. ATG7 is crucial for the elongation and closure of the autophagosome and for the conversion of LC3-I to its lipidated LC3-II form (58). In studies of JEV and autophagy, the key autophagy protein ATG7 is usually used as the target of autophagy. Knockdown of ATG7 in Neuro2a cells promotes JEV replication and viral yields (53). In contrast, the depletion of ATG7 in BHK-21 cells restricts JEV propagation (23). Previously, Huang et al. proved that JEV replication is restricted in ATG7 knockout A549 cell lines established with the CRISPR/Cas9 system (22). Consistently, we demonstrated that ATG7 knockdown and knockout caused remarkable decreases in viral titers in Huh7 cells. Depletion of the same gene has different effects on JEV replication, which may be related to the cell line. Taken together, our results suggest that JEV promotes its replication by activating autophagy in Huh7 cells.

Innate immunity is a basic defense that can be quickly called upon to fight the viral invasion. Autophagy affects the IFN-I response by regulating the expression of IFN-I and its receptor, and the cross talk between autophagy and the IFN-I response may be an important aspect of the molecular mechanism involving autophagy in innate antiviral immunity (32). IFN-I induces the ISG-dependent transcription of various proteins with antiviral effects (59). Here, we determined that Mx1 and ISG56 mRNA levels were further increased in ATG7-silenced cells. These data suggested that autophagy negatively modulated ISG activation.

Subsequently, we found that the knockdown of UAF1 promoted JEV-induced autophagy. Mx1 and ISG56 mRNA levels were reduced in UAF1-silenced cells. In the initial exploration, we screened many ISGs, such as IL-6, ISG15, IP-10, IL-1β, and so on, but UAF1 knockdown had a significant reduction on the level of Mx1 and ISG56. Flavivirus studies showed that Mx1 and ISG56 played an antiviral role (60). Simultaneously, we also explored IFN-β levels in UAF1 knockdown cells. Strikingly, IFN-β levels were not significantly changed in UAF1 knockdown cells. Transcription of ISGs occurs rapidly upon pathogen invasion, and this is classically provoked via activation of the JAK-STAT pathway, mainly by IFNs. However, a plethora of recent studies reported a variety of noncanonical mechanisms regulating ISG transcription (61). Whether the changes of Mx1 and ISG56 levels are affected by UAF1 through a nonclassical mechanism needs to be further explored. We speculated that the regulation of JEV replication by UAF1 was caused by autophagy. To verify this hypothesis, KO-ATG7 cells were transfected with UAF1 siRNA or NC siRNA to explore JEV replication and ISG levels. We found that the changes in JEV replication and ISG levels induced by UAF1 knockdown were blocked by autophagy. To confirm that ISGs participated in virus replication in a UAF1-driven manner, we overexpressed ISGs in UAF1 knockout cells. The results showed that ISGs suppressed JEV replication. Our results suggested that UAF1 modulated JEV replication through autophagy-induced ISG levels. More studies are needed to understand the details of UAF1-regulated autophagy.

In summary, this study clarified a novel function for UAF1 in regulating autophagy. In addition, the above data indicated that UAF1 modulates JEV replication through this newly identified function in regulating autophagy.

## MATERIALS AND METHODS

### Cells, viruses, and plasmids.

Huh7, HEK-293T, and BHK-21 cell lines were grown in Dulbecco’s modified Eagle’s medium (DMEM; Gibco) supplemented with 10% and 5% fetal bovine serum (FBS; Biological Industries, Israel) at 37°C in 5% CO_2_. The JEV strain SA14 (GenBank accession no. U14163) was kindly provided by Bo Zhang (Wuhan Institute of Virology, Chinese Academy of Sciences, China). The cDNA of human UAF1 was amplified by PCR and cloned into pRE-myc vectors (Key Laboratory of Zoonosis, Ministry of Agriculture and Rural Affairs, China). The UAF1 primers were used for plasmid amplification (5′ to 3′; forward, CTAGTCTAGAATGGCGGCCCATCAC; reverse, CCGCTCGAGCGTGGACTTCTGACGGTAA).

### Chemicals and antibodies.

Chloroquine (C129284) was purchased from Aladdin. Rapamycin (no. S1039) was obtained from Selleck Chemicals.

The antibody against LC3B (no. 2775) was purchased from Cell Signaling Technology. The antibodies against UAF1 (16503-1-AP), ATG7 (10088-2-AP), GAPDH (60004-1-lg), and SQSTM1 (18420-1-AP) were purchased from Proteintech. JEV E (GTX125867) was obtained from GeneTex. IRDye 800CW goat anti-rabbit IgG (926-32211) and IRDye 800CW goat anti-mouse IgG (926-32210) were purchased from LI-COR.

### Real-time RT-PCR.

Total RNA was extracted from the cells with a total-RNA kit (Fastagen, Shanghai, China) according to the manufacturer’s instructions. mRNA was synthesized to cDNA by using PrimeScript RT master mix (TaKaRa, Dalian, China) according to the protocol recommended by the manufacturer. Real-time PCR was performed using SYBR green PCR master mix (TaKaRa) on a Bio-Rad CFX96 Touch system (Bio-Rad, California, USA). The glyceraldehyde 3-phosphate dehydrogenase (GAPDH) gene was used as an internal control. The relative quantification of genes was analyzed using the 2^−ΔΔ^*^CT^* method. The data are the averages of results from three independent experiments performed in triplicate. The following primers were used (5′ to 3′): JEV E (forward, TCGGGAAGGGAAGCATTGAC; reverse, CTGTAAACTTTGCCGCCTGG), GAPDH (forward, TCAAGGCTGAGAACGGGAAG; reverse, TCGCCCCACTTGATTTTGGA), Mx1 (forward, AAGAGCCGGCTGTGGATATG; reverse, TTTGGACTTGGCGGTTCTGT), and ISG56 (forward, GTGCTTGAAGTGGACCCTGA; reverse, CCTGCCTTAGGGGAAGCAAA).

### Western blotting.

The cells were washed 3 times with cold phosphate-buffered saline (PBS) and incubated on ice with radioimmunoprecipitation assay (RIPA) buffer (Beyotime) containing a protease/phosphatase inhibitor cocktail (Beyotime) and phenylmethylsulfonyl fluoride (PMSF) (Beyotime) for 30 min. The lysates were centrifuged for 10 min at 4°C. Equal amounts of protein were denatured for 5 min in 5× SDS-PAGE loading buffer (Beyotime). The samples were electrophoresed and electrotransferred onto nitrocellulose membranes. Subsequently, the membranes were incubated with 5% nonfat dry milk for 2 h at room temperature and incubated with primary antibody and then secondary antibody. The membranes were visualized with an Odyssey system. Data from one experiment of three are shown. The target protein relative intensity, which was relative to GAPDH expression, was quantified using Image Studio V5.2.

### Generation of knockout cell lines.

Single guide RNAs (sgRNAs) targeting UAF1 and ATG7 were designed by Benchling (https://www.benchling.com/) and then cloned into plenti-CRISPRv2 (Addgene). HEK-293T cells were transfected with plenti-CRISPRv2-sgRNA, pSPAX2 (Addgene), and pMD2.G (Addgene) plasmids using Lipofectamine 2000 transfection reagent (Invitrogen) according to the manufacturer’s instructions. The supernatant containing lentivirus was collected at 48 hpt to infect Huh7 cells. These cells were selected with puromycin (1.5 μg/mL) for five passages. The effect of knockout was identified by Western blotting. sgRNAs are follows: sgUAF1, 5′-CACCGACATACCGAGTCCATGATGA-3′; sgATG7, 5′-CACCGGCCCGTTGCTGCCCAGCTAT-3′.

### siRNA transfection.

siRNAs targeting UAF1 (GGTCGAGACTCTATCATAA, GCAGAGATGTATAGCAACA, and GTATCAGGGTCCACTGAAA) and ATG7 (GAACGAGTATCGGCTGGAT, GATGTCGTCTTCCTATTGA, and ACTCGAGTCTTTCAAGACT) and a nontargeting control siRNA (siNC) were purchased from RiboBio. All the transfections were performed according to the manufacturer’s instructions using the transfection reagent Lipofectamine RNAiMAX (Invitrogen). Cells seeded in 12-well plates were transfected with siRNA (50 nM) mixed with Opti-MEM (Invitrogen) and incubated at 37°C to ensure the effectiveness of protein knockdown. The knockdown of protein levels was monitored by Western blotting.

### Autophagic flux detection.

Huh7 cells were infected with lentiviruses packaged with plenti-GFP-RFP-LC3, pSPAX2, and pMD2.G plasmids in HEK-293T cells. These cells were selected with puromycin (1.5 μg/mL) for five passages. Single clonal cells were collected by serial dilutions in 96-well plates.

GFP-RFP-LC3 Huh7 cells seeded on coverslips in 24-well plates were transfected with UAF1 siRNA or NC siRNA for 36 h and then mock infected or infected with JEV. Then, the cells were fixed and stained with DAPI at 24/36/48 hpi. Fluorescence signals were scanned under a Leica SP8 confocal microscope.

### Virus titer determination by the TCID_50_ method.

BHK-21 cells were seeded on 96-well plates. The cells were infected with a 10-fold series of the diluted viral medium. The cells were fixed with methanol at −20°C for 30 min at 48 hpi and detected by immunocytochemistry (62). The TCID_50_ was determined by using the method of Reed and Muench. The data were pooled from three experiments carried out in duplicate.

### Electron microscopy.

Huh7 cells were infected with JEV. The cells were fixed with 2.5% glutaraldehyde at 4°C for 1 h. The cells were collected and further fixed overnight. The cell pellets were dehydrated with an acetone series, embedded with epoxy resin, and cut into ultrathin sections. The images were observed with a JEM-2010 HR TEM.

### Statistical analysis.

The data were analyzed with Prism 7 software (GraphPad Software) using a two-tailed Student's *t* test.
